# Dataset of the role of green transformational leadership and individual green values in shaping firm reputation: The mediating effect of green supply chain management

**DOI:** 10.1016/j.dib.2026.112649

**Published:** 2026-03-07

**Authors:** Nicolay Alexander Galecio Sosa, Ruben Guevara

**Affiliations:** CENTRUM Catolica Graduate Business School, Lima, Peru – Pontificia Universidad Catolica del Peru, Peru

**Keywords:** Managers and large private companies in the food sector

## Abstract

This dataset was collected from a non-probability convenience sample of 359 managers from large private food sector companies in Lima, Peru, a strategic sector of the Peruvian economy characterized by the adoption of environmental management and sustainability practices. The dataset includes measures related to green transformational leadership, green supply chain management, individual green values, and firm reputation. The data are organized using standardized measurement scales and have been prepared to support advanced analytical techniques, including structural equation modeling. The dataset can be reused for (i) intersectoral comparative analyses, (ii) longitudinal studies on the evolution of green values and corporate reputation, (iii) validation and cross-cultural adaptation of measurement instruments, and (iv) multi-group analyses comparing organizational contexts, such as private versus public companies or different organizational sizes.

Specifications Table

**Table:** 

Subject	Earth & Environmental Sciences
Specific subject area	*Green Supply Chain Management.*
Type of data	Table, Image and Figure
Data collection	*Data collection was conducted in two stages: the first, during the second half of February and the first half of March 2020, and the second, during the second half of June and July 2020. During the data collection process, the authors personally contacted 500 respondents to request their participation in the study, obtaining a response rate of 71.80% (N = 359), which represents a robust response. All respondents answered the questions voluntarily* [1,2]*.* *The data were collected using a structured questionnaire administered to the study participants. The instrument allowed for the collection of information on the constructs analyzed and was designed to ensure clarity, consistency, and ease of response. Once collected, the data were reviewed, cleaned, and coded to guarantee their quality and coherence. Subsequently, they were organized into a structured dataset ready for use in various analytical approaches, allowing for their reuse in future studies* [3]*.*
Data source location	Lima Metropolitana, Peru.
Data accessibility	Repository name: Mendeley Data Data identification number: 10.17632/bs35yn7nkn.1 Direct URL to data: https://data.mendeley.com/datasets/bs35yn7nkn/ Galecio Sosa, Nicolay Alexander; Guevara, Ruben (2025), “The Role of Green Transformational Leadership and Individual Green Values in Shaping Firm Reputation: The Mediating Effect of Green Supply Chain Management”, Mendeley Data, V1, doi: 10.17632/bs35yn7nkn.1 [4]

## 1 Value of the Data

The data allow: (i) comparative analyses between industrial sectors, (ii) longitudinal research on the evolution of individual green values ​​and green transformational leadership, (iii) the application of multi-group models differentiating between public and private companies, and (iv) the replication and validation of measurement scales in different Latin American contexts. With these indications and clarifications, a clearer vision is offered of how the research community can reuse the dataset.

## 2 Background

As global sustainability challenges become more pressing, organizations face increasing pressure to incorporate environmental practices into their management processes to reduce negative impacts, comply with regulations, and strengthen their corporate reputation [5]. In this context, variables such as ecological transformational leadership, ecological supply chain management, individual ecological values, and corporate reputation have been extensively addressed in the organizational sustainability literature.

Several previous studies have used these variables to analyze environmental practices, organizational behaviors, and stakeholder perceptions in different economic sectors and geographic contexts [[6], [7], [8], [9], [10]]. However, access to structured datasets that integrate these variables, especially in emerging business contexts, remains limited.

The food sector is a particularly relevant environment for collecting this type of data, due to its environmental impact, economic importance, and exposure to international sustainability standards. In the case of large private food companies in Metropolitan Lima, Peru, many operate under strict regulatory requirements and hold recognized environmental certifications, such as ISO 14001, GlobalGAP, and HACCP, reinforcing the relevance of the analyzed context.

The presented dataset provides empirical information on managerial perceptions related to environmental leadership, environmental practices in the supply chain, individual environmental values, and corporate reputation. This data offers a useful basis for descriptive, comparative, and methodological studies, as well as for replicating analyses in other sectors or geographic contexts, contributing to future research on sustainability and business management.

## 3 Data Description

a) Data Type and Dataset Structure

The dataset consists of quantitative, survey-based information with a cross-sectional design. It comprises 359 observations, organized at the individual unit of analysis level. The data include variables related to green transformational leadership, green supply chain management, individual ecological values, and corporate reputation. The variables are measured perceptually and are numerically coded, facilitating statistical analysis. Measurements were taken using Likert-type scales, allowing for comparison and multivariate analysis of the evaluated constructs. b) Variable Description

The dataset includes four main variables:

Green Transformational Leadership: This represents leadership behaviors aimed at motivating and inspiring members of the organization to adopt environmentally responsible practices and support corporate sustainability goals [3].

Green Supply Chain Management: This encompasses organizational practices that integrate environmental criteria into supply chain activities, such as sourcing, production, distribution, and waste management [11].

Individual Green Values: These reflect the degree to which individuals possess personal values ​​and beliefs oriented toward environmental protection and sustainable behavior [3].

Firm Reputation: This expresses the general perception of stakeholders regarding the organization, particularly in relation to its environmental responsibility, ethical conduct, and sustainable performance [12].

The variables are organized into conceptual constructs, and each construct is composed of multiple observed indicators that represent specific dimensions of the study. c) Data Format and Availability

The dataset is provided in Excel, CSV, and SPSS formats and is structured in a tabular format, where rows represent individual observations and columns correspond to the variables of the different constructs.

The data is publicly available in the Mendeley Data repository (version 1), enabling its reuse, replication, and comparative analysis by other researchers. The repository includes organized Ydata files, as well as supplementary documentation that facilitates its understanding and implementation in different analytical environments.

Missing values ​​are clearly identified using a standardized coding scheme, ensuring transparency and consistency in the use of the dataset.

## 4 Experimental Design, Materials and Methods

a) Data type, Population and sample

The data were primary and original and were collected directly through a questionnaire administered to 359 managers of large private companies in the food sector in Metropolitan Lima, Peru.

In this research, the surveyed managers are part of large private companies in the food sector in Metropolitan Lima, Peru, which have international environmental management certifications, such as ISO 14001. This standard is recognized and accepted in Peru by the National Institute of Quality (INACAL) and is aligned with the General Environmental Law (Law No. 28611), which indicates the responsibility of private companies to prevent, mitigate, and remedy environmental impacts and degradation derived from their activities [13,14]. In this sense, ISO 14001 certification constitutes a solid and compelling reference criterion to justify that the selected organizations apply globally recognized environmental and sustainability practices.

The population consisted of 5,500 managers. In practice, we had direct access to a sample frame of 500, and 359 (71.8%) responded. Therefore, it corresponds to a non-probability convenience sample, since the complete list of the population was not available. Therefore, the sample obtained coincides with the statistical requirement (n=359, 95% confidence interval, and 5% error), which justifies the validity of the analysis performed.

$$\frac{n=N.Z^{2}.p.\;\left(1-p\right)}{e^{2}.\;\left(N-1\right)+Z^{2}.p.\;\left(1-p\right)}$$

Where:

• N = 5,500 (total managers: men and women).

• Z = 1.96 (95% confidence level).

• p = 0.5 (expected proportion).

• e = 0.05 (margin of error).

The quality indicators were as follows: Response rate: 71.80% (359 out of 500 surveys were completed correctly). Number of reminders sent: Two reminders were sent via email and phone call at 5-day intervals to encourage participation. Partial response rate: 2% (2 surveys were submitted incomplete and were excluded from the main analysis). Non-response rate: 26% (131 surveys were not answered).

The study sample included 291 males (81.1%) and 68 females (18.9%). The participants were managers and executives of both sexes who work in large private companies in the food sector in Lima, Peru that apply environmental and sustainability practices and that they were able to develop the questionnaires on topics of GTL, GSCM, IGV, and firm reputation. All targeted participants were: 337 managers (93.87%), 8 Directors (2.23%) and 14 Head/Chief/Boss (3.9%).

Table 1 shows the managerial level and gender of these managers. All constructs included in the conceptual model are reflective. b) Research Instruments

**Table 1:** Research sample characteristics.

Management level	Frequency	Proportion (%)	Cumulative %
Managers	337	93.87	93.87
Directors	8	2.23	96.1
Head/Chief/Boss	14	3.9	100
**Total**	**359**	**100**	

Gender	Frequency	Proportion (%)	Cumulative %
Male	291	81.1	81.1
Female	68	18.9	100
**Total**	**359**	**100**	

Note: The table presents the descriptive characteristics of the sample used in the study, including the relevant demographic and professional variables of the participants. These data help contextualize the profile of the analyzed population and support an appropriate interpretation of the model’s results.

This research obtained approval by an Ethics Committee prior to carrying it out. A non-probability convenience sample made up of 359 managers and executives of both sexes from large companies in the food sector in Lima, Perú [4] were chosen for this research [9].

The questionnaire was developed using the measurement scales of Zhou [2], Zhu [15], and Agyemang and Ansong [11], so that its items were clear, direct, and unambiguous, avoiding ambiguous or biased formulations. This reinforces the consistency and reliability of the responses collected for the research objectives.

GTL was measured with Zhou [2] scale, which includes 6 items and uses a 5-point Likert scale ranging from 1 (Strongly disagree) to 5 (Totally agree). GSCM was measured with Zhu [15] scale, which includes 21 items and uses a 5-point Likert scale ranging from 1 (Without considering it) to 5 (Implemented successfully). IGV was measured with Zhou [2] scale, which includes 9 items, and uses a 5-point Likert scale ranging from 1 (Strongly disagree) to 5 (Totally agree). Finally, the firm’s reputation was measured using Agyemang and Ansong [11] scale, which includes 5 items and uses a 5-point Likert scale ranging from 1 (Nothing) to 5 (To an excellent extent). Table A1 shows Scale Used to Measure the Research Variables.

c) Data Collection Procedure All participants signed an informed consent form after the research objectives were explained to them [12]. Participants were assured that their responses would be kept anonymous, so as to assure their confidentiality and that their information would be used to achieve the study purposes only [16]. The A non-probability convenience sample made up of managers and directors selected from large privates companies in the food sector [[6], [7], [8], [9], [10]]. During the data collection process, the authors contacted 500 respondents through personal contact, requesting their participation in the study, obtaining a response rate of 71.80% (N = 359), which is a strong response. All respondents answered the questions voluntarily [1,2].

It is indicated in the present study that the straight-line response patterns that exist in the dataset and that are part of or correspond to the way in which some respondents answered the questionnaire. d) Data preparation, coding, and quality checks

After data collection, responses were screened for completeness and consistency. Variables were coded and organized at the construct and indicator levels. Item grouping followed the conceptual definition of each construct, and variable labels were standardized to facilitate reuse. Missing values were identified using a standardized coding scheme. No inferential or model-based statistical analyses are reported in this data article. e) Dataset structure and potential reuse

The dataset is organized in a tabular format in which rows represent individual observations and columns correspond to observed indicators. The structure of the dataset allows users to apply a wide range of analytical techniques according to their research objectives, including descriptive, comparative, and multivariate approaches. The article does not report hypothesis testing, model estimation, or interpretation of relationships.

## Limitations

This dataset has some limitations that should be considered when reusing it. First, the information was collected from managers and executives of large private companies in the food sector located in Metropolitan Lima, Peru. Consequently, the data reflect perceptions specific to a particular organizational and geographic context, which may limit its direct applicability to other economic sectors, small and medium-sized enterprises, public sector organizations, or other national contexts.

Second, the data are based on self-reported responses obtained through surveys, and therefore may be influenced by participants' subjective perceptions and contextual factors present at the time of data collection. This should be considered by users of the dataset when conducting subsequent descriptive or comparative analyses.

Finally, although the dataset is structured at the level of constructs and indicators to facilitate its reuse, future researchers may apply additional data cleaning, filtering, or refinement procedures according to their specific research objectives and analytical approaches.

Future researchers using this dataset can apply additional filters or refinement criteria (e.g., exclusion of cases with low variance in responses), depending on the objectives of their analysis.

## Ethics Statement

This research obtained the approval by an Ethics Committee prior to carrying it out. This research obtained the approval by an Ethics Committee prior to carrying it out. The researchers declare that all participants received informed consent. All participants were systematically informed about the content and objectives of the study before participating. Participation was entirely voluntary. The study was conducted in accordance with the Declaration of Helsinki. For the data collection process, we submitted a statement to the participants from the Ethics Committee of CENTRUM Católica Graduate Business School, Lima, Peru – Pontifical Catholic University of Peru, dated October 21, 2019. In the statement, the Ethics Committee presents the researcher to facilitate the data collection process and indicates that the data collected are for academic purposes.

## CRediT Author Statement

**Nicolay Alexander Galecio Sosa:** Conceptualization, Data curation, Investigation, Methodology, Project administration, Resources, Visualization, Writing – original draft, and Writing – review and editing; **Ruben Guevara:** Supervision and Validation.

## Figures and Tables

**Table A1:** Scales Used to Measure the Research Variables.

Scales Used to Measure Green Transformational Leadership		
Item code	Item statement	Authors
GT1	The leader of the green product development project inspires the project members with the environmental plans.	[2].
GT2	The leader of the green product development project provides a clear environmental vision for the project members to follow.	
GT3	The leader of the green product development project gets the project members to work together for the same environmental goals.	
GT4	The leader of the green product development project encourages the project members to achieve the environmental goals.	
GT5	The leader of the green product development project acts with considering environmental beliefs of the project members.	
GT6	The leader of the green product development project stimulates the project members to think about green ideas.	

A five-point scale:1=strongly disagree, 2=disagree, 3= neither agree nor disagree, 4= agree, 5=totally agree.		

Scale Used to Measure Green Supply Chain Management		

GSCM1	Commitment to GSCM from senior managers	[15].
GCSM2	Support for GSCM from mid-level managers	
GCSM3	Cross-functional cooperation for environmental improvements	
GCSM4	Total quality environmental management	
GSCM5	Environmental compliance and auditing programs	
GCSM6	ISO 14001 certification	
GCSM7	Environmental management systems exist	
GCSM8	Eco-labeling of products	
GCSM9	Cooperation with suppliers for environmental objectives	
GCSM10	Environmental audit for suppliers’ internal management	

A five-point scale: 1=without considering it, 2=planning to consider it, 3=considering it currently, 4=starting implementation, 5: implemented successfully		

Scale Used to Measure Green Supply Chain Management		

GCSM11	Suppliers’ ISO 14000 certification	[15].
GSCM12	Second-tier supplier environmentally friendly practice evaluation	
GSCM13	Cooperation with customers for eco-design	
GSCM14	Cooperation with customers for cleaner production	
GSCM15	Cooperation with customers for green packaging	
GSCM16	Design of products for reduced consumption of material/energy	
GCSM17	Design of products for reuse, recycling, recovery of material, component parts	
GSCM18	Design of products to avoid or reduce use of hazardous products and/or in the manufacturing process	
GSCM19	Investment recovery (sale) of excess inventories/materials	
GSCM20	Sale of scrap and used materials	
GSCM21	Sale of excess capital equipment	
A five-point scale: 1=without considering it, 2=planning to consider it, 3=considering it currently, 4=starting implementation, 5: implemented successfully		
Scale Used to Measure Firm Reputation		
Item code	Item statement	Authors

FR1	Quality of products and services	[11].
FR2	Quality of staff	
FR3	Environmental responsibility	
FR4	Community responsibility	
FR5	Quality of management	

A five-point scale: 1=nothing, 2=to a small extent,3=to a medium extent, 4=largely to a large extent, 5=to an excellent extent.		

Scale Used to Measure Individual Green Values		

IGV1	I feel personally obliged to save as much energy as possible	[2].
IGV2	I feel morally obliged to save energy, regardless of what others do	
IGV3	I feel guilty when I waste energy	

A five-point scale: 1=strongly disagree, 2=disagree, 3= neither agree nor disagree, 4=agree, 5=totally agree		

Scale Used to Measure Individual Green Values		

IGV4	I feel morally obliged to use green instead of regular electricity	[2].
IGV5	People like me should do everything they can to reduce energy use	
IGV6	If I were to buy a new washing machine, I would feel morally obliged to buy an energy efficient one	
IGV7	I do not feel guilty at all when I buy vegetables and fruit from distant countries	
IGV8	I feel obliged to bear the environment and nature in mind in my daily behavior	
IGV9	I would be a better person if I saved energy	

A five-point scale: 1=strongly disagree, 2=disagree, 3= neither agree nor disagree, 4=agree, 5=totally agree		

*Note:* This table documents the items included in the dataset. No reliability, validity, or model evaluation results are reported in this data article. Item codes are provided for data documentation purposes. No factor analysis or measurement model evaluation is reported in this data article.

## Data Availability

Dataset of The Role of Green Transformational Leadership and Individual Green Values in Shaping Firm Reputation: The Mediating Effect of Green Supply Chain Management. (Original data).

## Appendix

The appendix includes scales used to measure multiple constructs: Green Supply Chain Management, Green Transformational Leadership, Firm’s Reputation, and Individual Green Values.
